# Bioremediation and Electricity Generation by Using Open and Closed Sediment Microbial Fuel Cells

**DOI:** 10.3389/fmicb.2018.03348

**Published:** 2019-01-14

**Authors:** Syed Zaghum Abbas, Mohd Rafatullah, Moonis Ali Khan, Masoom Raza Siddiqui

**Affiliations:** ^1^Division of Environmental Technology, School of Industrial Technology, Universiti Sains Malaysia, Penang, Malaysia; ^2^Chemistry Department, College of Sciences, King Saud University, Riyadh, Saudi Arabia

**Keywords:** bioremediation, exoelectrogens, power density, resistance, sediment microbial fuel cells

## Abstract

The industrial contamination of marine sediments with mercury, silver, and zinc in Penang, Malaysia was studied with bio-remediation coupled with power generation using membrane less open (aerated) and closed (non-aerated) sediment microbial fuel cells (SMFCs). The prototype for this SMFC is very similar to a natural aquatic environment because it is not stimulated externally and an oxygen sparger is inserted in the cathode chamber to create the aerobic environment in the open SMFC and no oxygen supplied in the closed SMFC. The open and closed SMFCs were showed the maximum voltage generation 300.5 mV (77.75 mW/m^2^) and 202.7 mV (45.04 (mW/m^2^), respectively. The cyclic voltammetry showed the oxidation peak in open SMFCs at +1.9 μA and reduction peak at -0.3 μA but in closed SMFCs oxidation and reduction peaks were noted at +1.5 μA and -1.0 μA, respectively. The overall impedance (anode, cathode and solution) of closed SMFCs was higher than open SMFCs. The charge transfer impedance showed that the rates of substrate oxidation and reduction were very low in the closed SMFCs than open SMFCs. The Nyquist arc indicated that O_2_ act as electron acceptor in the open SMFCs and CO_2_ in the closed SMFCs. The highest remediation efficiency of toxic metals [Hg (II) ions, Zn (II) ions, and Ag (I) ions] in the open SMFCs were 95.03%, 86.69%, and 83.65% in closed SMFCs were 69.53%, 66.57%, and 65.33%, respectively, observed during 60–80 days. The scanning electron microscope and 16S rRNA analysis showed diverse exoelectrogenic community in the open SMFCs and closed SMFCs. The results demonstrated that open SMFCs could be employed for the power generation and bioremediation of pollutants.

## Introduction

Toxic metals are released into the environment by many anthropogenic sources like discharge of municipal, agricultural, industrial, or residential waste products. The contamination of aquatic environment by heavy metals is of important concern due to accumulation of metals and their toxicity in aquatic habitats (Seebold et al., 1981). Toxic metals that are released into the aquatic environment are ultimately incorporated into the aquatic sediments to varying degrees. The accumulation of toxic metals in sediments has serious environmental connections for river water quality and for local communities. For example, sediment uses a diet source by many freshwater invertebrates and can be vulnerable to toxic metals bioaccumulation. This bioaccumulation can possibly harmful for many species especially top ranked at the food chain, like human, fish and birds. Additionally, the recovery of stream sediments and metal-contaminated river poses a serious liability to local users via remobilization of toxic metals from agricultural soil into crop (Sahoo et al., 2017).

The marine coastal area of Bayan Lepas, Penang, Malaysia is highly contaminated with toxic metals (Hg^2+^, Zn^2+^, and Ag^2+^) due to number of manufacturing industries. There are many methods have been used to treat these contaminated sediments, like ozonation, electrochemical degradation, and dredging. The huge cost and negative impact on aquatic ecosystem, stops the encouragement of these technologies. The natural degradation/decomposition of sediments also got much attention but rate is very slow due to lack of proper electron donor and acceptor. Therefore, efficient and new technologies are needed to provide proper electron donor and acceptor. A membrane-less sediment microbial fuel cell (SMFCs), with cathode in overlying aerobic water and anode buried in the anaerobic sediments, was usually employed to generate electricity from the reduction of inorganics and oxidation of organics across an external loading (Zhu et al., 2016; Lai et al., 2017). During this process, the anode exhibited bacteria transfer the electrons to the anode by oxidation of organic matters and reduction of inorganic matters and O_2_ reduced near the cathode by accepting the electrons from the anode (Abbas et al., 2017). Attributable to its special properties, an SMFC could be employed as a power source for instruments installed in lake and marine environments with maintenance-free operation (Reimers and Wolf, 2018). In addition, the SMFC could be used for in-situ sediment bioremediation due to the anode can act as electron acceptor for long term without addition of any chemical compounds and it could also be employed for detection of pollutants, specific DNA strands and biological oxygen demand (BOD). There are many advantages of SMFCs for sediment remediation with producing renewable energy: (1) for the natural bioconversion mechanisms, the electrode can provide a less aggressive, inexhaustible, clean and flexibly portable electron acceptor or donor, (2) SMFCs cause minimum distraction to the native aquatic habitat, (3) controllable electrochemical parameters can be easily monitored for the remediation processes (Bose et al., 2018). Recent advancements have been developed to operate the SMFCs in the parallel and vertical series to enhance the efficiency and SMFCs launching at large scale. The employment of SMFCs stacking model is brand new advancement for SMFCs launching at field level (Zhou et al., 2018).

Few studies have been reported about the remediation of these toxic metals by using SMFCs. Recently, it was found that toxic metals ions acted as electron acceptors of cathode reduction reaction to enhance power production by being reduced to metallic ionic forms (Li et al., 2017). The high efficiency of SMFC with toxic metals ions reducing cathode was linked with the higher over-potential of oxygen than that of toxic metals ions (Hsu et al., 2017). In the SMFC, toxic metals ions are easily transport to the both sides of cathode and anode, revealing that the impact of toxic metals ions on the SMFC efficiency become more complex due to the abrupt balance among biological effect of toxic metals on the microbes of anode and it’s works as an electron acceptor at the cathode (Chen et al., 2016). In this study, three toxic metals (Hg, Zn, and Ag) were selected as representatives of hazardous contaminants to investigate their synchronized remediation with power generation by membrane less cathode (aerated and non-aerated) SMFCs. The operated model of SMFCs had bigger size than previous SMFCs, so this model will lead to one step close to SMFCs scale-up. The SMFCs has great potential to employ at field level for sediment remediation because sediment is sink of all contaminants. These toxic metals were selected because these toxic metals have different redox potential and toxicity levels (Abourached et al., 2014). The Hg is more toxic while Zn and Hg are least toxic but as their concentration increased more than WHO and sediment standards then they will be highly toxic for aquatic ecology that directly affect the human health. The performance of SMFCs in the terms of power generation, bioremediation of toxic metals and its microbial diversity were explored.

## Materials and Methods

### Sampling

Sediment samples were taken from the Bayan Lepas industrial zone (N 5.3^o^: E 100.2^o^) of Pulau Pinang, Malaysia using a grab sampler. At this area, subsurface sediment (0–15 cm) were collected and then packed into polycarbonates container. All sediment samples were passed via a strainer through with the hole size of 0.2 cm to remove the gravel and coarse debris. Then they were used without any pre-treatment after the mechanically homogenization. All samples of sediments were chemically and physically characterized as shown in the Table 1.

**Table 1:** Physio-chemical properties of sediments sampled from Bayan Lepas, marine stream.

Parameters	Untreated sediments
Temperature (°C)	26–30
Color	Black
pH	5.0–8.5
Silt (%, w/w)	80.7 ± 70.5
Clay (%, w/w)	4.9 ± 2.7
Sand (%, w/w)	15.9 ± 4.8
Water contents (%, w/w)	55.7 ± 3.80
Carbon contents (%, w/w)	3.5 ± 1.7
Electrical conductivity (μS cm^-1^)	480 ± 6.30
Hg (II) ions (mg/kg)	0.913 ± 0.910
Zn (II ions (mg/kg)	1211 ± 1202
Ag (I) ions (mg/kg)	9.214 ± 9.112


### SMFC Construction and Operation

**FIGURE 1:**
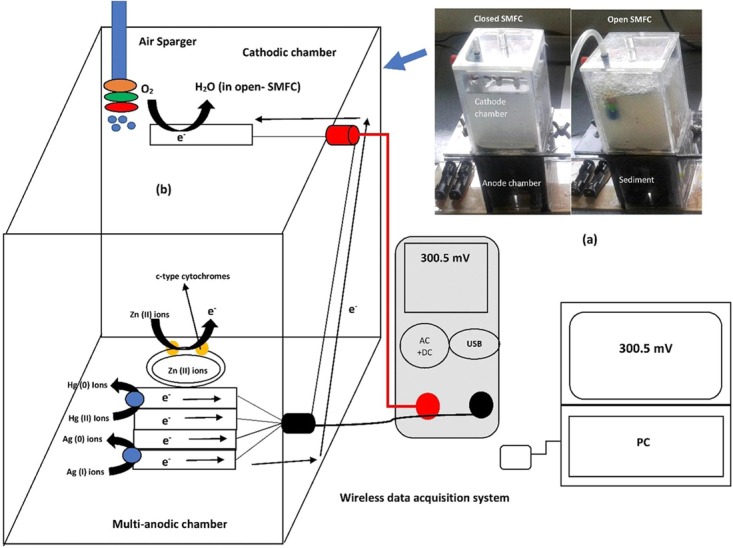
The lab-scale open and closed SMFCs designed models; schematic representation of operational SMFCs **(a)** and interaction of the toxic metals (Hg^2+^, Zn^2+^, and Ag^2+^) with electrodes attached biofilm and their remediation mechanisms synchronized with data wireless acquisition system **(b)**.

Membrane-less SMFC reactors were made of plexiglass in cylindrical shape. The height of SMFC was 176 mm and inner diameter 87 mm. Graphite felt electrodes (2 cm × 2 cm × 2 cm of thickness, GF series, FUDA 2B Lead, NY, United States) were used as both of cathode and anode with 70.5 cm^2^ of total projected surface area. The width and height of SMFCs were 8.7 cm and 17.6 cm, respectively. About 700 g wet sediment was loaded in each SMFC with 200 mL surface water in the cathode chamber. The nine pieces of anodes were buried horizontally in sediment and about 3 cm from the SMFC bottom and cathode was adjusted parallel in the overlying aerobic water surface exposed to air. The distance among cathode and anode was 4.0 cm. The platinum wires were tangled around the electrodes and anode and cathode were also connected by platinum wires. Two types of SMFC were operated. One was open SMFC (aerated) and second was closed SMFC (non-aerated) as shown in the Figure 1. During operation, the water loss through evaporation was accomplished with tap water to sustain a stable level of water.

### Electrochemical Measurements

The current and cell voltage between anode and cathode were automatically recorded at 1 min intervals using a data acquisition system (Extech instru, Model EX5422, FLIR systems, Inc., United States) until 120 days. The power density and polarization curves of SMFCs were obtained by connecting the cathode and anode electrodes to a variable resistance box (100 Ω–100 kΩ). The polarization curves were plotted to find the maximum power density with respect to optimum external resistance (Logan, 2015). The internal resistances were also calculated by using following formula and these values were obtained from polarization curve.

(1) ε=V+Ir

ε = electromotive force in volts, V

*I* = current in amperes, A

*R* = resistance of the load in the circuit in ohms, Ω

*r* = internal resistance of the microbial fuel cell in ohms, Ω

By arranging the equation (1) as following to calculate the internal resistances

(2) r=ε−V/I

At the maximum voltage, three conventional electrodes were used to obtain the cyclic voltammograms (CVs) of the anode (Metrohm, PGGSTAT12, Autolab, Echochemief). The cathode, anode and saturated calomel electrode (SCE) were used as counter electrode, working electrode and reference electrode, respectively. The SCE scan rate of 5 Vs^-1^ versus current range from -0.8 to +0.8 μA were used to measure the CVs of SMFCs. The sinusoidal perturbation signal of 5 V was used to analyze SMFCs internal impedances by measuring the electrochemical impedance spectroscopy (EIS).

### Toxic Metals Remediation

The concentrations of selected toxic metals (Hg, Zn, and Ag) were analyzed in the sediments samples before loading in the SMFCs by sequential extraction method using inductively coupled plasma mass spectrometry (Perkin Elmer-Sciex, model 250, United States). These concentrations were analyzed with the reference of Sediment Management Standards USA as shown in the Table 2. The toxic metals speciation before and after SMFCs operation were detected by X-Ray Photoelectron Spectroscopy (XPS) (Kratos Analytical, A Shimadzu group company, United Kingdom). The sediment samples were drawn after 20, 40, 60, 80, 100, and 120 days of operation from the SMFCs and measured the concentration of these metals by inductively coupled plasma mass spectrometry.

**Table 2:** The toxic metals removal capacities of open and closed SMFCs compared with Sediment Management Standards United States.

	Open SMFCs		Closed SMFCs		Days of operation	
				
Toxic metals	Concentrations (mg/kg)	Removal efficiency (%)	Concentrations (mg/kg)	Removal efficiency (%)		Sediment Management Standards United States
Hg (II) ions	1.941 ± 0.0	0	1.941 ± 0.0	0	0	0.41 mg/kg
	1.870 ± 0.002	3.640	1.890 ± 0.03	2.627	20	
	1.421 ± 0.002	26.75	1.670 ± 0.002	13.92	40	
	0.096 ± 0.001	95.03	1.219 ± 0.003	37.19	60	
	1.121 ± 0.001	42.22	0.591 ± 0.002	69.53	80	
	1.65 ± 0.003	14.99	1.412 ± 0.003	27.25	100	
	1.82 ± 0.002	6.233	1.874 ± 0.001	3.451	120	
Zn (II) ions	512.3 ± 0.0	0	512.3 ± 0.0	0	0	410 mg/kg
	350.4 ± 1.4	31.58	411.2 ± 3.9	19.72	20	
	178.5 ± 2.8	65.15	315.6 ± 2.7	38.38	40	
	68.16 ± 2.2	86.69	244.1 ± 2.4	52.34	60	
	256.8 ± 3.2	49.87	171.2 ± 3.3	66.57	80	
	346.2 ± 3.0	32.41	280.7 ± 3.1	45.20	100	
	481.1 ± 3.6	6.090	411.7 ± 3.3	19.63	120	
Ag (I) ions	10.51 ± 0.0	0	10.51 ± 0.0	0	0	6.1 mg/kg
	9.748 ± 0.4	7.514	10.00 ± 1.1	5.123	20	
	6.647 ± 0.9	36.93	8.318 ± 0.9	21.07	40	
	1.722 ± 0.4	83.65	6.876 ± 0.8	34.75	60	
	3.761 ± 0.8	64.31	3.653 ± 0.8	65.33	80	
	6.647 ± 0.4	36.93	8.201 ± 1.2	22.18	100	
	9.44 ± 0.4	10.43	9.008 ± 0.8	14.52	120	


### Biofilm Morphology of Electrodes

The scanning electron microscope (SEM) was employed to visualize bacterial biofilm attached to the electrodes. Electrodes were evacuated from the SMFCs boxes, washed with a sterile water, and for the fixing of samples immersed in 5% formaldehyde overnight. Then, for the dehydration of samples, graded solutions series of ethanol/water (25%, 50%, 75%, and 100%) and finally dried. The samples were then sputter coated with 10 nm Au/Pd (BAL-TEC SCD050; Bal-Tec) and seen in a Quanta 200 FEI, SEM (Sweden). The digitally SEM photos were obtained.

### DNA Extraction and 16s rRNA Gene Amplification

The anodes attached biofilms were scratched strenuously with sterilized razor to about 1 mm after 120 days and stored at -80°C prior to DNA extraction. The genomic DNA was extracted using Power-soil DNA extraction kit (Mo Bio Laboratories, Inc., United States). The NanoDrop spectrophotometer (Life Technologies/Thermo Fisher, Waltham, MA, United States) was used to measure the quality and concentration of extracted DNA. This extracted DNA was stored at -40^o^C after diluted to 10 ng μL^-1^ for downstream use.

The V1–V5 hypervariable region of the 16S rRNA genes were amplified from the extracted DNA with universal primers 515 F (5′-GTGCCGCMGCCGCGGTAA-3′) and 909 R (3′-CCCCGYCAATTCMTTTRAGT-5′) using a MiSeq sequencer. Each PCR reaction was contained 25 μL reaction mixture (TaKaRa, Dalian, China). About 1.0% agarose gel was used to electrophoresis of PCR products. The require band was purified by DNA gel extortion kit (Sangon Biotech, China) and stored at 4^o^C. The Illumina MiSeq system was used for the sequencing of purified amplicons. All recovered sequences were warily blasted using the Basic Local Alignment Search Tool (blastn) algorithm^1^.

## Results

### Voltage Generation From SMFCs

**FIGURE 2:**
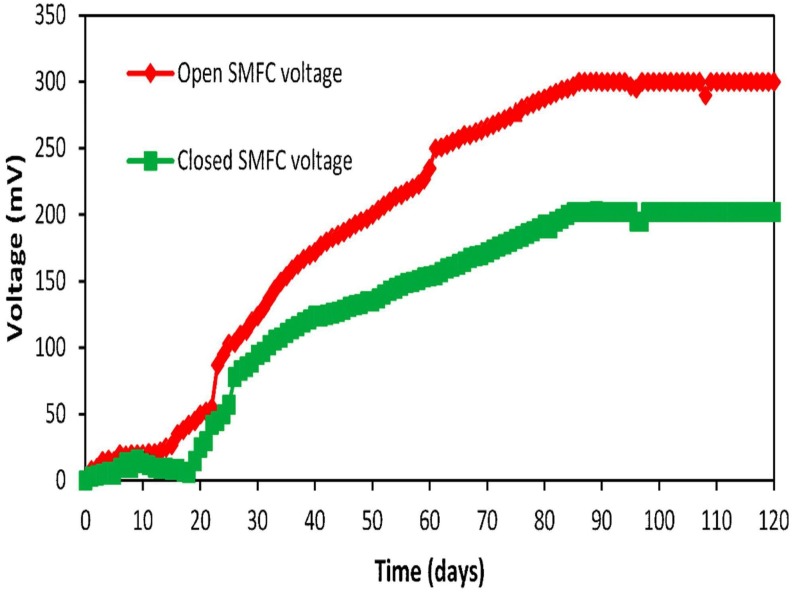
The average voltage generation comparison of open and closed SMFCs.

The open SMFCs showed the best average voltage between 60 and 80 days of operation as shown in the Figure 2. The active biofilm of exoelectrogens was developed on the electrodes surface during the starting reaction periods. After about 60 days, the voltage elevated and then dropped slowly. The average voltage of the open SMFCs (300.5 mV) was more than voltage of closed SMFCs (202.7 mV) between 60 and 80 days.

### Polarization Curves

**FIGURE 3:**
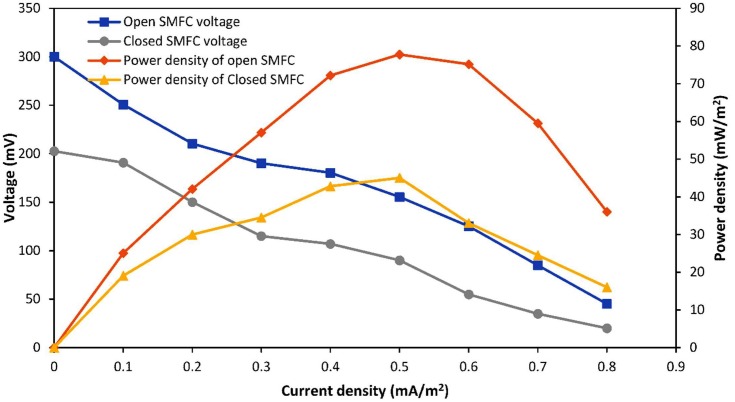
Polarization curves of open and closed SMFCs.

The polarization curves of SMFCs were used to calculate the optimal resistance systems. In this study, open SMFCs remained open at the start of operation to allow the voltage stabilize quickly. The external resistance value after 13 days in the open SMFCs was 250 Ω and optimal external resistance after 120 days was 1000 Ω. The optimal external resistance of open SMFCs was 1000 Ω with a maximum power density 77.75 mW/m^2^ as shown in the Figure 3. The internal resistances were calculated for both open and closed SMFCs by using this polarization curve. The internal resistance of open SMFCs was 981.5 Ω and for closed SMFCs was 478.5 Ω. The voltage lines with respect to current density were linear in the polarization curve so the internal resistances were almost close to optimum external resistances.

### Cyclic Voltammetry

**FIGURE 4:**
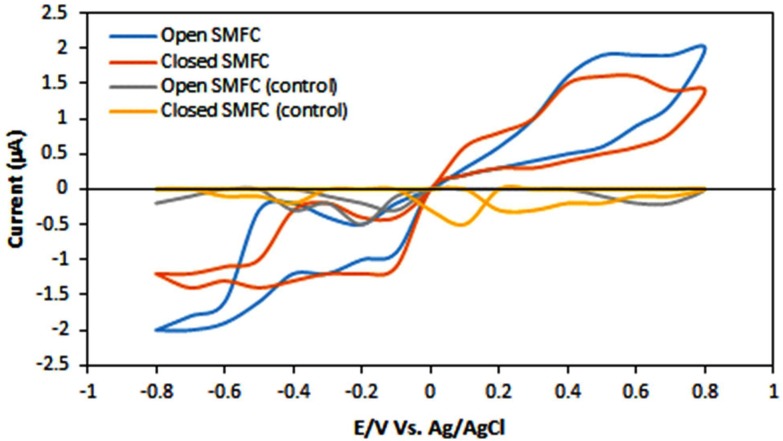
Cyclic voltammetry of open and closed SMFCs. The scan rate was 5 Vs^-1^. The dotted lines represent the oxidation and reduction peaks.

Cyclic voltammetry (CV) were conducted to indicate that power generation was connected to reduction and oxidation, carried by bacterial biofilms for power production. In CV analysis, the potential was slowly raised in forward scan from -0.8 μA to +0.8 μA, and the presence of current peak was due to reduction or oxidation in SMFCs. The voltage is then moved back to the initial potential. The CV analyses accounting colonization of electrodes (electrodes consisting a biofilm) in open and closed SMFCs presented the existence oxidation and reduction peaks as shown in the Figure 4, showing a current boost due to oxidation of sediment compounds that shifted electrons to the electrodes in the existence of a bacterial biofilm.

### Electrochemical Impedance Spectroscopy (EIS)

The EIS was studied to determine the three series resistances, anode (R), the solution (R), and cathode (R). It was observed that model of SMFC impedance spectra was reversible among cathode and anode. The estimation of individual impedance of anode and cathode were used to characterize the related impedance of these components. The analysis carried out for mature 120 days biofilms.

**FIGURE 5:**
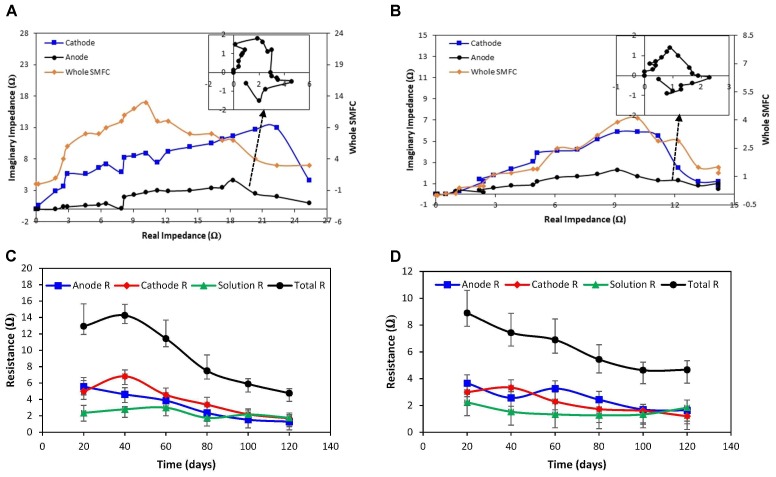
Nyquist curves for cathode, anode and whole SMFCs: **(A)** closed SMFCs and **(B)** open SMFCs. The inset represents the anode response. The behavior of anode, cathode, and solution impedances during embellishment of exoelectrogenic microbes in both SMFC over time: **(C)** closed SMFCs and **(D)** open SMFCs.

Electrochemical impedance of both open and closed SMFCs were shown in the Figures 5A,B. The inset semicircle represents the charge transfer rate of anode. The overall EIS of closed SMFCs was higher than open SMFCs. The anode charge transfer rate had more variation (closed SMFCs, 0.4 to -0.6 Ω: open SMFCs, 0.3 to -0.2 Ω) than cathode so it’s mean that charge transfer rate is highly depend on anode.

The values of anode (R), cathode (R), and solution (R) of SMFCs were illustrated in Figures 5C,D. The anode and cathode ohmic resistances were lower may be due to close proximity of reference electrode. The solution resistance almost same in all samples treated SMFCs. The cathodic impedance in the closed SMFCs was higher as compared to open SMFCs cathodes because of fast diffusion of oxygen at the cathode terminal than anode so it suggests that cathodic reactions are also limiting factor. In the closed SMFCs, the anodic impedance was higher than cathode because of dominance of anaerobic bacterial metabolism at that anode terminal.

#### EIS Response of SMFCs

**FIGURE 6:**
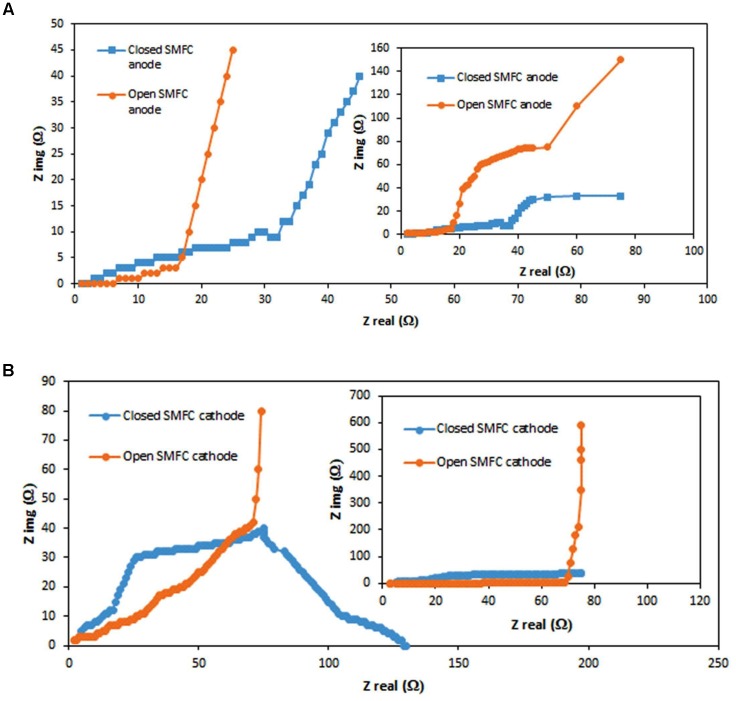
Nyquist plots for open and closed SMFCs anodes **(A)** and cathodes **(B)**. The overall frequency range from 10 kHz to 10 MHz shown in the inset graphs.

Electrochemical impedance spectroscopy evaluations of the open and closed SMFCs depend on the presence and absence of oxygen were recorded. Figures 6A,B represent the Nyquist plots for anode and cathode of both closed and open SMFCs. When CO_2_ acts as a terminal electron acceptor in the closed SMFCs, the Nyquist plots of anode (Figure 6A) and cathode (Figure 6B) were recognizable.

### Toxic Metals Bioremediation

**FIGURE 7:**
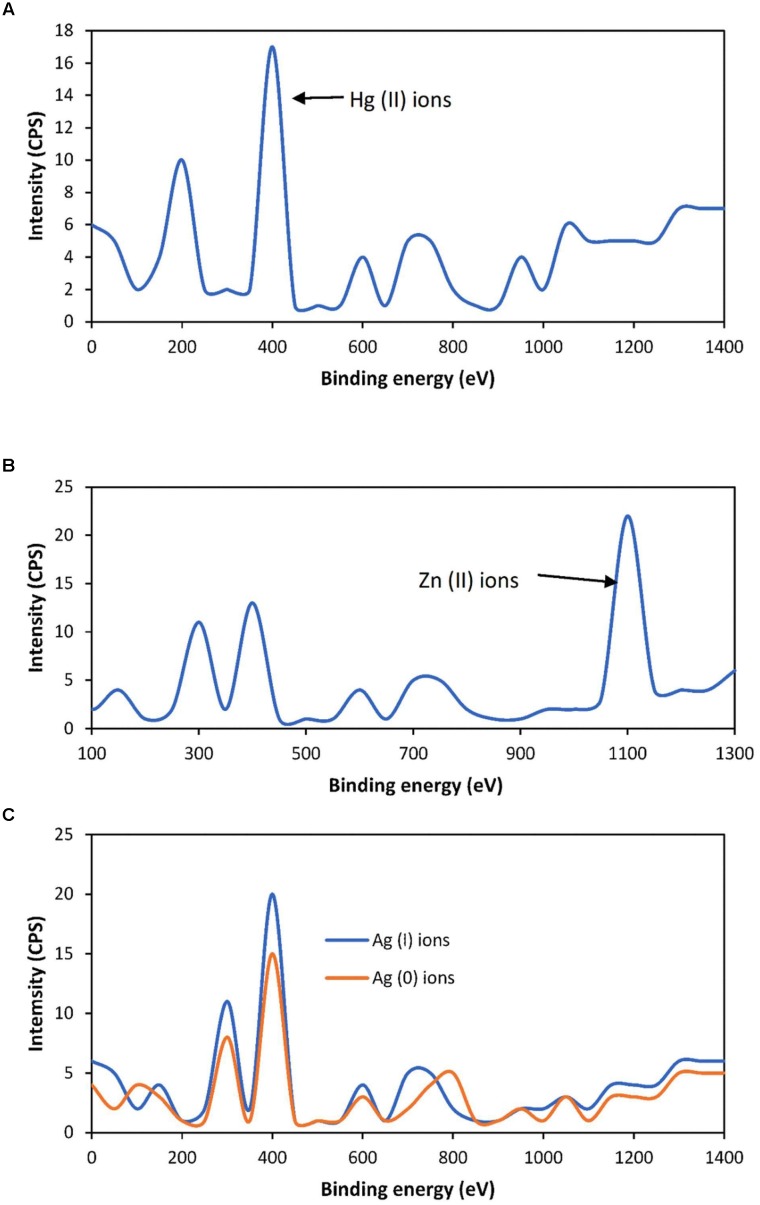
X-Ray Photoelectron Spectroscopy analysis of Hg (II) ions **(A)** Zn (II) ions **(B)**, and Ag (I) ions **(C)**.

The voltage was produced through transferring the electrons to the electrodes released from the reduction and oxidation of toxic metals at the anode terminal and moves to the cathode across the external load and thus the remediation of toxic metals was considered to be positively related with power generation. The highest removal of toxic metals in both SMFCs was noticed during 60–80 days and that removal level satisfy to the sediment guidelines. The toxic metals speciation were detected by XPS before and after SMFCs operation as shown in the Figures 7A–C. The optimum remediation of these toxic metals was measured during 60–80 days. The highest detoxification efficiency of Hg (II) ions, Zn (II) ions, and Ag (II) ions in the open SMFCs was 95.03%, 86.69%, and 83.65%, respectively. The threshold level of Hg (II) ions, Zn (II) ions, and Ag (II) reduction in the closed SMFCs was 69.53%, 66.57%, and 65.33%, respectively, as shown in the Table 2.

### Biofilm Morphology

**FIGURE 8:**
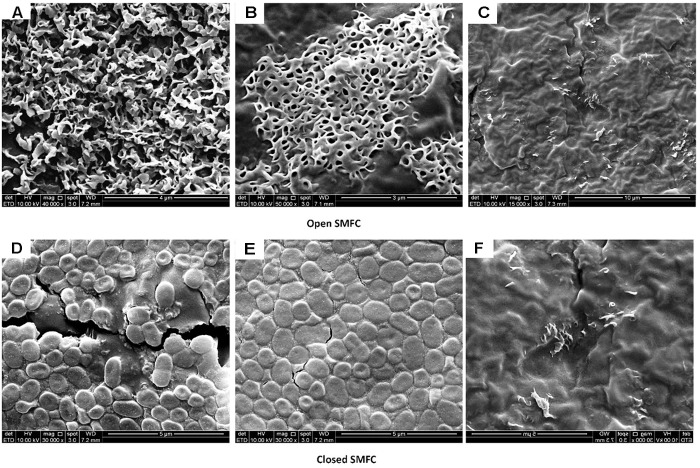
The digital images captured by scanning electron microscope of open and closed SMFCs: **(A,D)** anode, **(B,E)** cathode, and **(C,F)** control.

The difference in the morphology of electrodes biofilms of open and closed SMFCs is illustrated in the Figure 8 representing the electron microscopy images of biofilm formation on carbon graphite felt with the electrochemically active biofilms. A dense and thick biofilm on graphite rod can be observed, but the biofim structure is different where the biofilm is colonizing on each carbon fiber with thickness of 3–4 μm. There is more space available among intersections of carbon fiber biofilm being compact and dense biofilm colonies on carbon rod. This may provide easy substrate contact resulting in high current density. For a bioelectrochemical system, the carbon material may be more suitable due to high access of exoelectrogens. However, there are many other factors like liability, needed space to clogging and cost of the electrode material that have to be taken into more investigation.

### Bacterial Community Analyses

There is large difference between the anodes attached bacterial communities of both SMFCs as shown in the Table 3. The open SMFCs dominated by *Pseudomonas* strains, *Geobacter sulfurreducens, Clostridium sp. P2T, Azovibrio restrictus S5b2, Dechloromonas* sp. *CLT* and *Azospirillum* sp. *Mat2-1a.* The presence of *Geobacter sulfurreducens* enhanced the power generation because it is most effective and highly electrogenically active bacteria. In the closed SMFCs, *Sedimentibacter saalensis* have been detected which has the ability to remove the toxic metals under anaerobic conditions, however, its role in the electro-active biofilm is still unknown.

**Table 3:** Phylogenetic identification of anodic exoelectrogens diversity of open and closed SMFCs.

Accession number	Name of bacteria	Similarity index (%)
**Open SMFCs**		
FJ652606	*Pseudomonas fluorescens*	100
GSU13928	*Geobacter sulfurreducens*	99
AY949856	*Clostridium* sp. P2T	100
AF011346	*Azovibrio restrictus* S5b2	100
AF170354	*Dechloromonas* sp. CLT	99
AY118222	*Azospirillum* sp. Mat2-1a	100
AB246809	*Pseudomonas boreopolis*	87
**Closed SMFCs**		
AJ493052	*Desulfosporosinus orientis*, DSMZ 7493	100
AM084020	*Comamonas* sp. R-25060	99
AY178844	*Treponema* sp. Sy24	99
NR_026515.1	*Aeribacillus pallidus* strain DSM 3670	100
AJ242495.1	*Thermicanus aegyptius* ET-5b	100
AF138734.1	*Thermoactinomyces intermedius KCTC 9646*	100
AY631277.1	*Thermincola ferriacetica Z-0001*	100
AJ404680	*Sedimentibacter saalensis*	100


## Discussion

This high amount of voltage in open SMFCs may be due to the impact of aeration in the open SMFCs. Similar voltage with infinite resistance was obtained by Logan in the open SMFCs (Logan, 2008). Zhang et al. (2011) obtained about 770–870 mV by using open SMFCs voltage sediment of Lake Michigan. The time of voltage stabilization vary in every SMFC and largely depend upon the biofilm formation on the anode surface. In this study, the voltage was stabilized after 14 days in the open SMFCs. The voltage stabilization in the open SMFCs after 13 days was also reported by An et al. (2010). The voltage stabilization in the closed SMFCs was observed after 20 days. This may be due to the late adjustment of exoelectrogens to the SMFCs environment.

Previous study by González-Gamboa et al. (2017) reported the 0.6 V with 1000 Ω after 120 days of open SMFCs operation because during this phase, exoelectrogens deliver the maximum electron to the electrodes. In the closed SMFCs, the external resistance after 13 days was 10 Ω and optimal external resistance after 120 days was 500 Ω. Hong et al. (2010) were also reported 450 Ω resistance in the closed SMFCs with 0.2 V after 120 days of operation. Dumas et al. (2007) were also reported the 98 mW/m^2^ value obtained by modified graphite electrodes with Ni^2+^ and Mn^2+^ which function well at laboratory scale but fragile at field operation. The maximum power density of closed SMFCs was 45.04 mW/m^2^ with 500 Ω. Ryckelynck et al. (2005) found maximum power density about 11 mW/m^2^ with 10 cm of anode depth in the sediment but in this study, the depth of anode in the sediment was about 4 cm that is the main cause of high power density in this closed SMFCs. The reason behind the lower power densities of open SMFCs than closed SMFCs may be the permeability of oxygen in anodic chamber will results into use of anodic electron for oxygen in anodic chamber and hence it will be loss for current production. Furthermore, the voltage losses (activation and ohmic over-potential) in open SMFCs may be lower than closed SMFCs.

The oxidation peak was observed in the open SMFCs at +1.9 μA (forward scan) was due to oxidation of the sediment compounds. The reduction peak in open SMFCs was noted at -0.3 μA (reverse scan) and this peak was considered to emerge from breakdown of the products like metabolites as glucose (added in both SMFCs to maintain bacterial metabolism) and different mediators (flavin and cytochrome). When CV was operated under the vacancy of oxygen conditions, the little oxidation peak was observed at +1.5 μA (forward scan) reduction peak was observed at -0.3 μA (reverse scan). This is may be due to the anaerobic degradation of toxic metals. These CV results showed the higher biofilm electro-activity in open SMFCs than closed SMFCs. The existence of this redox peak may be indicates the presence of electrons mediators produced by exoelectrogens that are reversibly reduced in the CV scans (Zou et al., 2017). However, the amplitude of peak is smaller (0.5–1.5 V) than previously reported by Kim et al. (2007) about 50–150 V where it was demonstrated on the behalf of experiments that mediators were not important for electricity generation. Thus, we can say that mediators produced by exoelectrogens are crucial for the degradation of toxic metals in the SMFCs.

When oxygen used as electron acceptor cathode of open SMFCs showed higher Nyquist arc than cathode of closed SMFCs because the catalyst influence mass transfer and charge transfer ultimately overall impedance. The properties of catalyst (biofilm variation and cultivation time) also highly influence the impedance of SMFCs (Wang et al., 2018).

From EIS results, it can be concluded that overall charge transfer resistance decreased at anode. This results in low anode activation loss (Jia et al., 2018) also concluded that electron transfer at the anode effect the overall bio-electrochemical reaction. Though, the charge transfer rate not only depend upon the extracellular microbial electron-transfer activity but also influenced by anodic biofilms.

It can be concluded that this can be referred to the anode terminal for CTI of the electron redox shuttle mechanisms. The rate of substrate oxidation was very low in the closed SMFC which lead low CTI, indicated by the Nyquist arc with low frequency, boost the electron kinetics shift to anode from substrate. The investigations are persistent with transfer of charge mechanism in which substrate oxidation consider as the rate inhibiting point (Agostino et al., 2017).

Since reduction of CO_2_ is a fast process, the impedance was very low related with charge transfer so Nyquist plots was not showing any extension in the low frequency region. By contrast, bio-electrochemical rates of oxidation at the anode are kinetically reluctant. The high transfer rate and extension in the low frequency region was shown by Nyquist plots similar with the previous studies (Li et al., 2016). On the other side, when oxygen acted as the terminal acceptor of electrons, low frequencies shown by onset of a second Nyquist arc at the cathode. The second arc attributes to the larger impedance for reduction of oxygen on graphite felt contrasted to the CO_2_ reduction (Doyle et al., 2017). This impedance behavior also influenced by many factors like mass transfer, charge transfer and catalytic effects. The biocatalysts properties (microbes, oxygen) of both closed and open SMFCs were different so the impedance behavior of both anodes were not identical to each other in this study. Nevertheless, overall anode impedance at any given ac frequency for both SMFCs were comparable.

Some researchers reported that the highest removal of toxic metals in SMFCs was between 20 and 40 days of SMFCs operation in the pure culture of exoelectrogens due to the early adjustment with surrounding environment (Hong et al., 2010). The mixed culture of exoelectrogens normally require more time to adjust with surrounding environment due to intraspecific or interspecific competition (Morris and Jin, 2012). The exoelectrogens reduced the Hg (II) ions to less soluble and non-toxic Hg (0) ions but these ions are volatile ions and can’t be detected by XPS. Wang et al. (2011) were successfully removed the Hg (II) ions with power generation by using MFCs. They reported that after 10 h reaction, the concentration of Hg (II) ions in the effluent was in the range of 0.44–0.69 mg/L with maximum power generation (433.1 mW/m^2^) at pH 2.0. The Zn (II) ions were bio-accumulate inside the cell. Abourached et al. (2014) were removed about 97% of Zn (II) ions with simultaneous highest power generation about 3.6 W/m^2^ via single-chamber MFCs. The exoelectrogens also reduced the soluble and toxic Ag (I) ions to less soluble and non-toxic Ag (0) ions. Choi and Cui (2012) were removed the Ag (I) ions with simultaneous power generation by using cost-effective MFCs. They reported that after 8 h reaction, the maximum Ag (I) ions removal was 99.91 ± 0.00–98.26 ± 0.01% with maximum voltage (0.749 V), maximum power (4.25 W/m^2^) and maximum current density 5.67 (A/m^2^) at load resistance of 1000 Ω. The high value of toxic metals removal in the open SMFCs than closed SMFCs was due to the rapid reduction of oxygen in the cathode chamber. It’s also depend on the type of microorganisms, sediment residence time hydraulic residence time (Neethu and Ghangrekar, 2017).

The remediation mechanisms of these toxic metals with power generation were shown in the Figure 1B. The Hg (II) ions are normally attached to the exoelectrogens periplasm through *MerP* protein and then transported inside the cell by *MerT* transporter. The intercellular reduction of Hg (II) ions to elementals, volatile and non-toxic Hg (0) ions was performed by mercuric reductase (*MerA*). After reduction, the Hg (0) ions were efflux to the outside of the cell (Jadán-Piedra et al., 2017). The Zn (II) ions are normally attached with c-type cytochromes of exoelectrogens like *OmcA* and *OmcZ* and reduced these cytochromes and finally accumulate inside the cell (Carpio et al., 2016). The Ag (I) ions attach with the periplasmic Ag (I)-binding protein (SilE) encoded by gene *silE*. This protein reduced the Ag (I) ions to elemental and non-toxic Ag (0) ions (Raheem et al., 2016). The mechanisms of toxic metals remediation in both open and closed SMFCs are same but the rate of remediation was different due to the acting of O_2_ as electron mediator for exoelectrogens in the open SMFCs. The O_2_ is gaseous requirement for mostly bacteria which enhance the biodegradation of toxic metals (Najafabadi et al., 2016).

These filamentous structures in the open SMFCs might simply have been main cause of electrons transfer and more resistant to toxic metals (Xu et al., 2017a), but their high growth did not show to disturb power generation. The capabilities of filamentous bacteria for extracellular electron transfer have been reported in few studies. These filamentous bacteria has a potential to employ for the removal of toxic metals fom sediment by using SMFCs. One of the main problem of biofilm SMFCs is clogging, particularly when the substrate is sediment. These filamentous bacteria are resistant to hydraulic shock so promptly clogging happen which halt biofilm reconstruction (De Sá et al., 2017). This clogging problem can be easily solve by using the open SMFCs with contineous supply of oxygen. The low performance of closed SMFCs may be due to the presence of rod-shaped bacteria because the rod-shaped bacteria are not resistant to the hydraulic shock, easily dispersed and also lack of electrons conductiong wires like pili (Abbas et al., 2017).

The 1,000-fold lower conductivity of *Geobacter* pili is enough for the effective cell to cell electron transfer (Rojas et al., 2017). The *Geobacter* spp. were detected in many previous MFCs studies (Erable et al., 2017). The *Clostridium* spp. also reported to act as exoelectrogens (Jiang et al., 2016). Almost all clones in the open SMFCs showed 99–100% similarity index except *Pseudomonas boreopolis*. The *Azovibrio restrictus* have been detected in this studies but the exact role of this bacteria in the biofilm is still unknown. The detection of *Desulfosporosinus orientis* also gives clue about its syntrophic interaction among exoelectrogens active bacteria and fermentative bacteria (Schilirò et al., 2016). *Thermicanus aegyptius, Thermoactinomyces intermedius*, and *Thermincola ferriacetica* are strictly anaerobes, fermentative microbes and could degrade many organic and inorganic compounds. They consumed the organic and inorganic compounds for boosting their growth (Xu et al., 2017b).

## Conclusion

This study demostrates the comparion of open and closed SMFCs performance. The results indicate that open SMCFs showed better performance than closed SMFCs. Thus, open SMFCs is a promising tool to enhance the power generation and can promote environmental standard by reducing the environmentals contaminants. This study also shows the divergence of microorganisms which are responsible for power generation and pollutants removal. The SMFCs is a good option to restore the organic and inorganic compounds. It is hoped that this research contributes to some productive knowledge into the field application of SMFCs too some extent.

## Author Contributions

MR and SA designed the experiments and wrote the manuscript. SA was responsible for performing all experiments. MK and MS analyzed the data. All authors contributed to interpreting the results, critically revising the manuscript for important intellectual content, and approving the final manuscript.

## Conflict of Interest Statement

The authors declare that the research was conducted in the absence of any commercial or financial relationships that could be construed as a potential conflict of interest.

## Footnotes

1 http://www.ncbi.nlm.nih.gov/BLAST

## References

[B1] Abbas S. Z. Rafatullah M. Ismail N. Syakir M. I. (2017). A review on sediment microbial fuel cells as a new source of sustainable energy and heavy metal remediation: mechanisms and future prospective. *Int. J. Energy Res.* 41 1242–1264. 10.1002/er.3706

[B2] Abourached C. Catal T. Liu H. (2014). Efficacy of single-chamber microbial fuel cells for removal of cadmium and zinc with simultaneous electricity production. *Water Res.* 51 228–233. 10.1016/j.watres.2013.10.062 24289949

[B3] Agostino V. Ahmed D. Sacco A. Margaria V. Armato C. Quaglio M. (2017). Electrochemical analysis of microbial fuel cells based on enriched biofilm communities from freshwater sediment. *Electrochim. Acta* 237 133–143. 10.1016/j.electacta.2017.03.186

[B4] An J. Lee S.-J. Ng H. Y. Chang I. S. (2010). Determination of effects of turbulence flow in a cathode environment on electricity generation using a tidal mud-based cylindrical-type sediment microbial fuel cell. *J. Environ. Manage.* 91 2478–2482. 10.1016/j.jenvman.2010.06.022 20688427

[B5] Bose D. Bose A. Mitra S. Jain H. Parashar P. (2018). Analysis of sediment-microbial fuel cell power production in series and parallel configurations. *Nat. Environ. Pollut. Technol.* 17 311–314.

[B6] Carpio I. E. M. Franco D. C. Sato M. I. Z. Sakata S. Pellizari V. H. Ferreira Filho S. S. (2016). Biostimulation of metal-resistant microbial consortium to remove zinc from contaminated environments. *Sci. Total Environ.* 550 670–675. 10.1016/j.scitotenv.2016.01.149 26849331

[B7] Chen S. Tang J. Fu L. Yuan Y. Zhou S. (2016). Biochar improves sediment microbial fuel cell performance in low conductivity freshwater sediment. *J. Soils Sediments* 16 2326–2334. 10.1007/s11368-016-1452-z

[B8] Choi C. Cui Y. (2012). Recovery of silver from wastewater coupled with power generation using a microbial fuel cell. *Bioresour. Technol.* 107 522–525. 10.1016/j.biortech.2011.12.058 22217729

[B9] De Sá J. Mezzomo H. Fraga M. Ogrodowski C. Santana F. (2017). Anode air exposure during microbial fuel cell operation inoculated with marine sediment. *J. Environ. Chem. Eng.* 5 1821–1827. 10.1016/j.jece.2017.03.024

[B10] Doyle L. E. Yung P. Y. Mitra S. D. Wuertz S. Williams R. B. Lauro F. M. (2017). Electrochemical and genomic analysis of novel electroactive isolates obtained via potentiostatic enrichment from tropical sediment. *J. Power Sources* 356 539–548. 10.1016/j.jpowsour.2017.03.147

[B11] Dumas C. Mollica A. Féron D. Basséguy R. Etcheverry L. Bergel A. (2007). Marine microbial fuel cell: use of stainless steel electrodes as anode and cathode materials. *Electrochim. Acta* 53 468–473. 10.1016/j.electacta.2007.06.069 19534134

[B12] Erable B. Byrne N. Etcheverry L. Achouak W. Bergel A. (2017). Single medium microbial fuel cell: stainless steel and graphite electrode materials select bacterial communities resulting in opposite electrocatalytic activities. *Int. J. Hydrog. Energy* 42 26059–26067. 10.1016/j.ijhydene.2017.08.178

[B13] González-Gamboa N. K. Valdés-Lozano D. S. Barahona-Pérez L. F. Alzate-Gaviria L. Domínguez-Maldonado J. A. (2017). Removal of organic matter and electricity generation of sediments from Progreso, Yucatan, Mexico, in a sediment microbial fuel cell. *Environ. Sci. Pollut. Res.* 24 5868–5876. 10.1007/s11356-016-8286-5 28063086

[B14] Hong S. W. Kim H. S. Chung T. H. (2010). Alteration of sediment organic matter in sediment microbial fuel cells. *Environ. Pollut.* 158 185–191. 10.1016/j.envpol.2009.07.022 19665268

[B15] Hsu L. Mohamed A. Ha P. T. Bloom J. Ewing T. Arias-Thode M. (2017). The influence of energy harvesting strategies on performance and microbial community for sediment microbial fuel cells. *J. Electrochem. Soc.* 164 H3109–H3114. 10.1149/2.0171703jes

[B16] Jadán-Piedra C. Alcántara C. Monedero V. Zúñiga M. Vélez D. Devesa V. (2017). The use of lactic acid bacteria to reduce mercury bioaccessibility. *Food Chem.* 228 158–166. 10.1016/j.foodchem.2017.01.157 28317709

[B17] Jia Y.-H. Qi Z.-L. You H. (2018). Power production enhancement with polyaniline composite anode in benthic microbial fuel cells. *J. Central South Univ.* 25 499–505. 10.1007/s11771-018-3754-3

[B18] Jiang Y.-B. Zhong W.-H. Han C. Deng H. (2016). Characterization of electricity generated by soil in microbial fuel cells and the isolation of soil source exoelectrogenic bacteria. *Front. Microbiol.* 7:1776. 10.3389/fmicb.2016.01776 27877168 PMC5099896

[B19] Kim J. R. Jung S. H. Regan J. M. Logan B. E. (2007). Electricity generation and microbial community analysis of alcohol powered microbial fuel cells. *Bioresour. Technol.* 98 2568–2577. 10.1016/j.biortech.2006.09.036 17097875

[B20] Lai C.-Y. Liu S.-H. Wu G.-P. Lin C.-W. (2017). Enhanced bio-decolorization of acid orange 7 and electricity generation in microbial fuel cells with superabsorbent-containing membrane and laccase-based bio-cathode. *J. Clean. Prod.* 166 381–386. 10.1016/j.jclepro.2017.08.047

[B21] Li X. Mu S. Ren Y. Wang X. (2017). Behavior of copper in membrane-less sediment microbial fuel cell. *J. Renew. Sustain. Energy* 9:023103 10.1063/1.4979569

[B22] Li X. Wang X. Zhao Q. Zhang Y. Zhou Q. (2016). In situ representation of soil/sediment conductivity using electrochemical impedance spectroscopy. *Sensors* 16:625. 10.3390/s16050625 27144567 PMC4883316

[B23] Logan B. E. (2008). *Microbial Fuel Cells.* Hoboken, NJ: John Wiley & Sons.

[B24] Logan B. E. (2015). *Microbial Fuel Cell Technologies for Renewable Power and Biofuels Production From Waste Biomass.* Available at: http://hdl.handle.net/1853/53187

[B25] Morris J. M. Jin S. (2012). Enhanced biodegradation of hydrocarbon-contaminated sediments using microbial fuel cells. *J. Hazard. Mater.* 213 474–477. 10.1016/j.jhazmat.2012.02.029 22402341

[B26] Najafabadi A. T. Ng N. Gyenge E. (2016). Electrochemically exfoliated graphene anodes with enhanced biocurrent production in single-chamber air-breathing microbial fuel cells. *Biosens. Bioelectron.* 81 103–110. 10.1016/j.bios.2016.02.054 26926591

[B27] Neethu B. Ghangrekar M. (2017). Electricity generation through a photo sediment microbial fuel cell using algae at the cathode. *Wat. Sci. Technol.* 76 3269–3277. 10.2166/wst.2017.485 29236006

[B28] Raheem H. Q. Al-Thahab A. A. Abd F. G. (2016). Different methods for detection sliver nanoparticles produced by *Proteus mirabilis* bacteria. *Int. J. Pharm. Technol. Res.* 9 368–376.

[B29] Reimers C. E. Wolf M. (2018). Power from benthic microbial fuel cells drives autonomous sensors and acoustic modems. *Oceanography* 31 98–103. 10.5670/oceanog.2018.115

[B30] Rojas C. Vargas I. T. Bruns M. A. Regan J. M. (2017). Electrochemically active microorganisms from an acid mine drainage-affected site promote cathode oxidation in microbial fuel cells. *Bioelectrochemistry* 118 139–146. 10.1016/j.bioelechem.2017.07.013 28803164

[B31] Ryckelynck N. Stecher H. A. Reimers C. E. (2005). Understanding the anodic mechanism of a seafloor fuel cell: interactions between geochemistry and microbial activity. *Biogeochemistry* 76 113–139. 10.1007/s10533-005-2671-3

[B32] Sahoo P. Tripathy S. Panigrahi M. Equeenuddin S. M. (2017). Anthropogenic contamination and risk assessment of heavy metals in stream sediments influenced by acid mine drainage from a northeast coalfield. India. *Bull. Eng. Geol. Environ.* 76 537–552. 10.1007/s10064-016-0975-2

[B33] Schilirò T. Tommasi T. Armato C. Hidalgo D. Traversi D. Bocchini S. (2016). The study of electrochemically active planktonic microbes in microbial fuel cells in relation to different carbon-based anode materials. *Energy* 106 277–284. 10.1016/j.energy.2016.03.004

[B34] Seebold I. Labarta C. Amigó J. (1981). “Heavy metals in the sediments,” in *Analytical Techniques in Environmental Chemistry 2: Proceedings of the Second International Congress, Barcelona*, ed. Albaiges J. (Spain: Elsevier).

[B35] Wang C.-T. Sangeetha T. Yan W.-M. Chong W.-T. Saw L.-H. Zhao F. (2018). Application of interface material and effects of oxygen gradient on the performance of single-chamber sediment microbial fuel cells (SSMFCs). *J. Environ. Sci.* 75 163–168. 10.1016/j.jes.2018.1003.1013 30473281

[B36] Wang Z. Lim B. Choi C. (2011). Removal of Hg2+ as an electron acceptor coupled with power generation using a microbial fuel cell. *Bioresour. Technol.* 102 6304–6307. 10.1016/j.biortech.2011.02.027 21377357

[B37] Xu P. Xiao E.-R. Xu D. Zhou Y. He F. Liu B.-Y. (2017a). Internal nitrogen removal from sediments by the hybrid system of microbial fuel cells and submerged aquatic plants. *PLoS One* 12:e0172757. 10.1371/journal.pone.0172757 28241072 PMC5328281

[B38] Xu P. Xiao E. Xu D. Li J. Zhang Y. Dai Z. (2017b). Enhanced phosphorus reduction in simulated eutrophic water: a comparative study of submerged macrophytes, sediment microbial fuel cells, and their combination. *Environ. Technol.* 39 1–14. 10.1080/09593330.2017.1323955 28443365

[B39] Zhang F. Tian L. He Z. (2011). Powering a wireless temperature sensor using sediment microbial fuel cells with vertical arrangement of electrodes. *J. Power Sources* 196 9568–9573. 10.1016/j.jpowsour.2011.07.037

[B40] Zhou C. Fu Y. Zhang H. Chen W. Liu Z. Liu Z. (2018). Structure design and performance comparison of large-scale marine sediment microbial fuel cells in lab and real sea as power source to drive monitoring instruments for long-term work. *Ionics* 24 797–805. 10.1007/s11581-017-2251-2

[B41] Zhu D. Wang D.-B. Song T.-S. Guo T. Wei P. Ouyang P. (2016). Enhancement of cellulose degradation in freshwater sediments by a sediment microbial fuel cell. *Biotechnol. Lett.* 38 271–277. 10.1007/s10529-015-1985-z 26543037

[B42] Zou L. Qiao Y. Zhong C. Li C. M. (2017). Enabling fast electron transfer through both bacterial outer-membrane redox centers and endogenous electron mediators by polyaniline hybridized large-mesoporous carbon anode for high-performance microbial fuel cells. *Electrochim. Acta* 229 31–38. 10.1016/j.electacta.2017.01.081

